# Essential Oil-Based Bioherbicides: Human Health Risks Analysis

**DOI:** 10.3390/ijms22179396

**Published:** 2021-08-30

**Authors:** Chloë Maes, Jeroen Meersmans, Laurence Lins, Sandrine Bouquillon, Marie-Laure Fauconnier

**Affiliations:** 1 Institut de Chimie Moléculaire de Reims, UMR CNRS 7312, Université Reims-Champagne-Ardenne, UFR Sciences, BP 1039 boîte 44, CEDEX 2, 51687 Reims, France; chloe.maes@uliege.be (C.M.); sandrine.bouquillon@univ-reims.fr (S.B.); 2 Laboratoire de Chimie des Molécules Naturelles, Gembloux Agro-Bio Tech., Université de Liège, 5030 Gembloux, Belgium; 3 TERRA Teaching and Research Centre, Gembloux Agro-Bio Tech., Université de Liège, 5030 Gembloux, Belgium; jeroen.meersmans@uliege.be; 4 Laboratoire de Biophysique Moléculaire aux Interfaces, Gembloux Agro-Bio Tech., Université de Liège, 5030 Gembloux, Belgium; l.lins@uliege.be

**Keywords:** essential oils, human health, biopesticides, bioherbicide, risks

## Abstract

In recent years, the development of new bio-based products for biocontrol has been gaining importance as it contributes to reducing the use of synthetic herbicides in agriculture. Conventional herbicides (i.e., the ones with synthetic molecules) can lead to adverse effects such as human diseases (cancers, neurodegenerative diseases, reproductive perturbations, etc.) but also to disturbing the environment because of their drift in the air, transport throughout aquatic systems and persistence across different environments. The use of natural molecules seems to be a very good alternative for maintaining productive agriculture but without the negative side effects of synthetic herbicides. In this context, essential oils and their components are increasingly studied in order to produce several categories of biopesticides thanks to their well-known biocidal activities. However, these molecules can also be potentially hazardous to humans and the environment. This article reviews the state of the literature and regulations with regard to the potential risks related to the use of essential oils as bioherbicides in agricultural and horticultural applications.

## 1. Introduction

As the awareness of the risks related to the overuse of synthetic pesticides and their hazardous consequences (off-targeted toxicities and high persistence) is growing, a lot of ongoing research focuses on the potential of natural molecules as an alternative for biocontrol [1]. It induces the opposition of two types of chemical pesticides: the first one, more conventional, is the use of synthetic molecules with all the well-known side effects, and the second one is the use of natural molecules mainly extracted from plants and with potentially less risks. The integrated pest management (IPM) and integrated weed management (IWM) strategies are considered eco-friendly agronomic practices for pest/weed control. When it comes to weed control, four types of action are possible, i.e., (i) physical (crop rotation, cover cropping, interrow hoeing, thermal method, etc.), (ii) chemical (use of bioherbicides), (iii) mechanical and (iv) biological (use of microorganisms). The use of allelopathy for weed control is also a strategy within IWM. This method consists in weed control by release of secondary metabolites (called allelochemicals) by crops into the environment. It is very useful in combination with methods cited previously [2]. Among plant secondary metabolites used as biopesticides, essential oils (EOs) are a main class used in a growing number of applications (herbicides, insecticides, fungicides, bactericides, acaricides) [3,4,5,6]. Indeed, lots of EOs have shown phytotoxic effects, which can be promoted by their use as bioherbicides [7,8]. However, even if these compounds are natural, it is equally important to study their risks for human health and the environment [9,10]. This article focuses on the human health risks of EOs used as bioherbicides in an agronomic and horticultural context. Among all herbicides of which hazards have been studied, a huge need for less harmful herbicides exists since glyphosate has been banned in many countries [11,12,13,14]. After presenting some generalities about pesticides, risks and EOs, this paper provides a critical analysis of the risks to human health associated with the use of EO-based bioherbicides. In this respect, attention is directed to how they are assessed as well as the current status of research.

### 1.1. Generalities about Pesticides

A pesticide is a product used to control all types of pests (insects, weeds, fungi, bacteria or mites) in agriculture, animal breeding and public health [15]. They can be used in a wide range of cases: the principal is to protect agriculture, but they are also used to prevent any human health risks (giant hogweed, tiger mosquito, etc.), to avoid damage to infrastructure (ivy: Hedera helix; termites) or to control invasive exotic species (e.g., Asian hornet) [16]. This implies many different modes of application and uses, which obviously involve various risks as well. For the rest of the discussion, we will focus on pesticides used for the protection of plants, called plant protection products (PPPs) [7].

#### 1.1.1. Plant Protection Products

PPPs contain at least one active substance and are used in various sectors (agriculture, horticulture, gardens, public areas and forestry). The active substance is the component with a proven direct or indirect biological activity. It can be a synthetic (in conventional pesticides) or a natural substance (in biopesticides) and is regulated by authorities [17]. The wide use of PPPs has contributed to the green revolution: crop yields increased from 1 ton per hectare (T/Ha) in 1960 to 5 T/Ha in 2013. [18]. In agronomy, they are used for plant disease management (the main categories dealing with this are fungicides and insecticides), but also for weed control and soil preparation [19,20]. When considering the last two applications, they are defined as herbicides.

#### 1.1.2. Herbicide Mechanisms of Action

Herbicides are used in various cases: pre- or post-emergence, with a selective effect or not. Indeed, depending on the molecule action mode, the herbicide will work on some types of plants (selective) or all of them (non-selective) and/or on different stages of growth.

An active substance can act on one or more metabolic pathways which are characterized by phytotoxic effects. Conventional herbicides can affect multiple processes, such as photosynthesis, biosynthesis of amino acids or the intracellular redox potential of targeted plants [20,21].

As an illustration, glyphosate inhibits an enzyme (5-enolpyruvylshikimate-3-phosphate synthetase) by acting on manganese chelation [22]. This results in multiple phytotoxic effects of which the inhibition of the biosynthesis of aromatic amino acids (shikimate pathway) is the most important. Thereby, as only transgenic glyphosate-resistant crops are not affected, this is a non-selective effect [21]. Another molecule, metribuzin, is a pre- and post-emergent selective herbicide (against a range of di- and monocot weeds) which inhibits photosynthesis at the level of Hill’s reaction, blocking electron transport, leading to lipid and chlorophyll photooxidation [23]. Some sulfonylureas, including rimsulfuron, are selective herbicides that affect the biosynthesis of branched-chain amino acids by influencing the enzyme acetolactate synthase [21].

Pelargonic acid (nonanoic acid), obtained by chemical synthesis but found in its natural version in *Pelargonium*, is a non-selective herbicide gaining popularity in both conventional and organic agriculture because of its broad-spectrum contact action [24]. Indeed, these middle-chain fatty acids cause severe damage to cell membranes and degrade linolenic acid in the thylakoid membranes, inducing a strong and rapid electrolyte leakage [25].

When considering bioherbicides, the active substance must be a natural extract or molecule. Some plant extracts (including molecules such as sorgoleone, artemisinin, ailanthone, sarmentine, pelargonic acid and juglone; chemical groups such as triketones, catechins, quinones, alkaloids and polyacetylenes; or complex blends such as essential oils) are allelochemicals with bioherbicide effects [26]. For EOs, the mechanism of action will be discussed below.

Regardless of the types of herbicide or their mode of action, these are chemicals that must be regulated for the desired and unwanted effects to be put to good use.

#### 1.1.3. Pesticide Regulations

Pesticides are regulated by different legislations depending on the region of the world, such as the European Commission (EC, European Union, EU), Environmental Protection Agency (EPA, Washington, USA), Department of Health (Canberra, Australia), Institute for the Control of Agrochemicals under the Ministry of Agricultural and Ministry of Agricultural (Beijing, China), Government of Japan, Food Safety and Standards Authority of India and three federal ministries of Brazil [27,28,29,30]. Some international organizations exist to help countries with pesticide management, such as the International Code of Conduct on the Distribution and Use of Pesticide, created in 1985 by the Food and Agriculture Organization. However, major complications persist at the international level because of the non-harmonization of quantities of maximum residue levels and the legislation stringency gap between developing and developed nations [31].

From a European point of view, the use of PPPs is regulated by the regulation (EC)1107/2009 in cooperation with some other European regulations and directives. For the approval and authorization of pesticides, a two-tiered approach is used, i.e., firstly, the approbation of the pesticide active substance by the EU, and secondly, the procedure of approval of the PPP. Once it is approved, a monitoring program will be set up by the European Food Safety Authority [7].

All the countries mentioned above have developed regulations with the same purposes. In the USA, the tolerance reassessment and registration review program has been established by the EPA. In Australia, a national harmonization has been implemented, whereas in China and India, the regulatory systems are still poorly enforced [31].

As pesticides aim to destroy pests, they have toxicity for some organisms, but a major drawback is that this toxicity may also affect non-targeted organisms (for example, soil microfauna or pollinator insects) [32].

### 1.2. Definition of “Risk”

The risk results from the combination of the hazard generated by a product and the exposure of humans to this product. Considering herbicides used to protect crops before and after harvest from infestation by pests and plant diseases, several human health risks exist and are typically induced by (i) the usage of the product (direct health risk of the herbicide applicant) and (ii) the presence of herbicide residues in feed or in nature. In essence, after the application, herbicides may be distributed into the environment following different pathways, i.e., by the air (drift), through aquatic systems (runoff, drainage) and through absorption by humans and biota, including the targeted pest itself [33]. All these different pathways of herbicide distribution need to be considered in the approval procedure of PPPs. In order to decrease the hazard, the production of bioherbicides, which are based on natural active substances and therefore potentially less toxic molecules (such as EOs), is promoted.

### 1.3. Generalities about Essential Oils (EOs)

Essential oils are a complex mixture of volatile compounds obtained by distillation of different parts of aromatic plants, or by cold expression for citrus EOs. The molecules composing EOs are mainly terpenes (monoterpenes and sesquiterpenes), terpenoids and phenylpropanoids. They are secondary metabolites of plants produced by three biosynthetic pathways (mevalonate, methyl-erythritol and shikimic acid) [8]. Terpenes are composed of isoprene units (five-carbon base) which can be functionalized, substituted or rearranged to create acyclic, monocyclic, bicyclic or tricyclic forms of carbure, alcohol, aldehyde, ketone, ester, ether, peroxide and phenol functions. Monoterpenes are composed of two isoprene units, and sesquiterpenes are composed of three [34]. Terpenoids are terpenes containing oxygen. Phenylpropanoids are derived from phenylpropane and comprise some aldehydes, alcohols, phenols, methoxy derivatives and methylene dioxy compounds [35].

Each EO is a combination of ten to several hundreds of these molecules in variable proportions which results in a very complex composition. Each compound can have one or multiple biological activities, whereas some associated interaction may strengthen (synergy) or weaken (antagonism) these. EOs are used in beverage, food, perfume, cosmetics and, more recently, agronomic applications. Based on their biocidal activities, they are good candidates for the development of new bioherbicides [8,10]. EO compositions can vary following genetic differences (chemotypes, species, parts of plants), but also by different environmental conditions (time of harvest, soil composition, climate) [8]. For this reason, it is better to consider the toxicity of each component individually and then consider that of EOs depending on the precise composition of the targeted batch.

Considering herbicidal effects, the already known effective compounds are β-pinene, farnesene, eugenol, 1,8-cineole (eucalyptol), juglone, α-pinene, camphor, limonene, pulegone, menthol, menthone, citral, carvacrol, R/S-carvone, trans-caryophyllene, thymol, geraniol and citronellol [7]. All EOs containing these compounds have a herbicidal activity depending on their concentration. Other EOs showing a herbicidal activity, probably due to synergy of their constituents, are: *Cymbopogon citratus*, *Hyptis suaveolens*, *Artemisia fragrans*, *Origanum vulgare*, *Citrus aurantiifolia*, *Plectrantus amboinicus*, *Mentha longifolia*, *Nepeta nuda*, *Salvia leucophylla*, *Vitex negundo*, *Syzygium aromaticum*, *Mentha x piperita*, *Cinnamomum zeylanicum*, *Cymbopogon winterianus*, *Pogostemon benghalensis*, *Monarda didyma*, *Artemisia scoparia*, *Heterothalamus psiadioides* [36].

In addition, some compounds of EOs are effective as germination inhibitors: borneolcamphor, menthol, β-citronellol, (R)-carvone, citral, geraniol, limonene, menthone, α-carvacrol. Some others reduce the radicle length: borneol, camphor, β-citronellol, (R)-carvone, carvacrol, thymol and limonene [37].

Some EOs have effects on all plants, whereas others are effective only on certain ones. This specificity can come from the mechanism of action of the active constituent.

The mechanisms of action of some EOs and their constituents are as follows [7,26]:

1. To disrupt the cuticle and cause desiccation or burning of young tissues (constituents: 1,8-cineole, 2-acetonaphthone and 3-isothujone);
2. To target photosynthesis and mitochondrial respiration inhibition (EOs: *Cymbopogon citratus*, *Hyptis suaveolens*, *Artemisia fragrans*, *Origanum vulgare*; constituents: β-pinene, farnesene, eugenol, 1,8-cineole, juglone, α-pinene, camphor, eucalyptol, limonene, pulegone, menthol, menthone);
3. To change enzymatic and phytohormone regulation (EOs: *Mentha x piperita*; constituents: R/S-carvone, farnesene);
4. To alter water status (EOs: *Syzygium aromaticum*; constituents: camphor, menthol, eugenol, citral, trans-caryophyllene);
5. To alter membrane properties and interactions (EOs: *Mentha piperita*, *Cinnamomum zylanicum*, *Cymbopogon winterianus*; constituents: 1,8-cineole, thymol, menthol, geraniol, camphor);
6. To induce microtubule disruption and genotoxicity (EOs: *Citrus aurantiifolia*, *Plectrantus amboinicus*, *Mentha longifolia*, *Nepeta nuda*, *Salvia leucophylla*, *Vitex negundo*; constituents: citral, limonene, carvacrol, pulegone, menthone, S-carvone);
7. To induce reactive oxygen and nitrogen species (EOs: *Psilanthus benghalensis*, *Monarda didyma*, *Artemisia scoparia*, *Heterothalamus psiadioides*; constituents: α-pinene, β-pinene, citronellol).

The two major advantages of the usage of EOs as herbicides, compared to synthetic molecules, are (i) their high volatility, considerably decreasing the residues in the soil, food or water, and (ii) their high complexity of composition, decreasing the possibility to develop weed resistance as several modes of action can be present [38].

## 2. Risks of the Use of EOs as Bioherbicides

As mentioned above, the risk is the combination of hazard and exposure. In this review, we will focus on human health risks when EOs are used as PPPs, specifically, as bioherbicides, even if we recognize the importance of the risks to the environment and, as such, non-target organisms including soil microorganisms, aquatic fauna and beneficial insects. We will first assess all damage that EOs can cause to human health [33] and, subsequently, discuss the specific risks depending on the associated rates of exposure (e.g., contact, ingestion, inhalation or injection). Two types of herbicide poisoning are possible: acute (high-dose, single-event exposure) vs. chronical (low-dose, long-term exposure) [33]. The measuring tool is the median lethal dose (LD50) which represents the amount of toxic substance producing a 50% mortality of the tested organisms, under controlled conditions for a time of 24 h, expressed in milligrams of herbicide per kilogram of animal weight (mg/kg) [39,40].

### 2.1. Hazard Identification for EOs

As EOs have biological activities that allow pest control [41], there is also a chance that these activities affect human health. Over recent decades, research has highlighted the neurotoxicity, genotoxicity and fertility and mutagenic impacts of conventional herbicides, resulting in a wide range of symptoms. Symptoms and diseases such as headache, fatigue, nausea, skin irritation, respiratory illness, cardiovascular illness, gastroenteritis and enhanced risk of cancer and Parkinson’s disease have been documented [1]. The most common hazardous effects observed for components and EOs are summarized in Table 1. Among these, known herbicide molecules have been underlined. For components where it was available, the LD50 (mg/kg) for rodents is added in brackets.

#### 2.1.1. Cytotoxicity

Cytotoxicity has been found in vitro for EOs in Gram-positive and -negative bacteria, in deoxyribonucleic acid (DNA) or ribonucleic acid (RNA) viruses and in fungi, including yeasts. Several action modes have been identified such as permeabilization of the membranes, coagulation of the cytoplasm or lipid and protein damages. These effects lead to leakage of macromolecules and to lysis [8,48]. In eukaryotic cells, a depolarization of the mitochondrial membranes occurs (affecting Ca^++^ cycling) which induces a chain reaction leading to cell apoptosis and necrosis. In particular, phenols, aldehydes and alcohols cause these effects [41].

Although these effects are used to protect humans, animals and agriculture from pathogens, some major components of EOs have shown a cytotoxic effect in mammalian cells in vitro through the induction of apoptosis and necrosis. Unscheduled DNA synthesis (UDS) tests, allowing for detecting genotoxicity, have been performed on some phenols of EOs and found that eugenol, isoeugenol, methyleugenol and safrole induce cytotoxicity and genotoxicity in rodent hepatocytes, while estragole (main compound of *Artemisia dracunculus* EO and present in *Ocimum basilicum* EO) induces UDS in hamster fibroblastic V79 cells [35,38].

A special type of cytotoxicity, which occurs only when the molecules are exposed to light (particularly ultraviolet radiation) is called phototoxicity. A study focusing on the murine fibroblastic cell line 3T3 and the rabbit cornea-derived cell line SIRC (Statens Seruminstitut Rabbit Cornea) concluded that *Citrus aurantium dulcis* and *Cymbopogon citratus* EOs were phototoxic and cytotoxic. However, this study also found that *Fusanus spicatus* wood EO was not phototoxic but was very cytotoxic (as it induces damage to the cellular and organelle membranes and acts as prooxidant on proteins and DNA) [46].

#### 2.1.2. Mutagenicity and Carcinogenicity

In general, EOs and their main components do not seem to induce nuclear mutations of living organisms. However, some exceptions have been illustrated, such as several monoterpenes and alkenylbenzenes having nuclear mutagenicity potency in mammals [42]. In the Ames test (bacterial carcinogenic test with high correlation results for animals), some EO components (menthone, anethol, asarone, trans-anethole oxide, trans-asarone oxide, terpineol, cinnamaldehyde, carvacrol, thymol and carvone) showed mutagenic effects. In addition, other tests showed the induction of cancers in some cases: for example, the *Drosophila melanogaster* somatic mutation and recombination test (SMART) is positive for menthone; the mouse lymphoma assay (MLA) is positive for anethol; and the sister chromatid exchange (SCE) test is positive for asarone [35]. Finally, eugenol induces chromosomal aberration and endoreduplications in rabbit V79 cells (mutagenic effect) [49].

Similarly, the majority of EOs are devoid of carcinogenicity, but some of their major components can be considered as secondary carcinogens after metabolic activation. As an illustration, (i) *Salvia sclarea* and *Melaleuca quinquenervia* EOs induce estrogen secretion that can lead to estrogen-dependent cancers, (ii) psoralen, flavins, cyanine and porphyrin are photosensitizing molecules which can induce skin cancer and (iii) pulegone, safrole, methyleugenol, D-limonene and estragole induce some specific carcinogenic metabolites in rodents [35,43].

#### 2.1.3. Allergenic Effect

More than seventy-nine EOs have been reported for causing at least one type of contact allergy (i.e., allergic contact dermatitis). EOs responsible for the highest frequency of allergies are those from *Melaleuca alternifolia* (tea tree), *Cananga odorata* (ylang-ylang), *Lavendula augustifolia* (lavender), *Mentha x piperita* (peppermint), *Geranium*, *Rosa damascene* (rose), *Pinus* (turpentine) and *Satanlum* (sandalwood) [44]. Twenty-nine compounds of EOs are considered as allergens by the European Commission, i.e., limonene, linalool, estragole, phenyl acetaldehyde, methyl octinoate, citronellol, geraniol, benzyl alcohol, neral, geranial, α-isomethyl ionone, methyl eugenol, hydroxy citronellal, α-ionone, eugenol, cinnamaldehyde, vanillin, coumarin, benzyl benzoate, benzyl salicylate and benzyl cinnamate [45].

Major cases of allergic contact dermatitis have been reported for groups of people that have intensive contact with EOs (e.g., bar workers, citrus fruit pickers, hairdressers, cosmetics workers and aromatherapists) [50,51,52]. In the case of oral ingestion of EOs, a large amount of EOs (for example, 6–10 g of camphor) can be very dangerous and lead to coma [8]. However, less severe health issues are more common, including a burning sensation in the mouth and throat, nausea, vomiting and diarrhea. This does not prevent essential oils from being used in the food sector at lower doses (1.5 mg/person/day for camphor) without undesirable effects. The maximum oral and dermal doses vary depending on the EO’s major compounds [53].

On the contrary, some studies showed no negative impact of EOs on allergic asthma. For instance, no impact on lung function and methacholine responsiveness (use in asthma diagnosis) was noted after the exposure of twenty-five patients with allergic asthma twice a day for 4 weeks to a purifying air spray containing forty-one EOs [54,55].

#### 2.1.4. Reproductive Toxicity

The effect of EOs on pregnancy is a highly controversial matter. As it has been stated before, the composition of EOs will vary depending on lots of factors, meaning they do not always have the same effect on unborn children [56]. If we consider, rather, the EO components individually, some have been shown to modulate reproductive hormones and to have fetotoxic, abortifacient, embryotoxic and antigestational effects. These associated components are anethole, apiole, citral, camphor, thymoquinone, trans-sabinyl acetate, methyl salicylate, thujone, pulegone, β-elemene, β-eudesmol and costus lactone, among others [47].

### 2.2. Risk for Human Health Related to the Use of EO Herbicide Products

**Figure 1:**
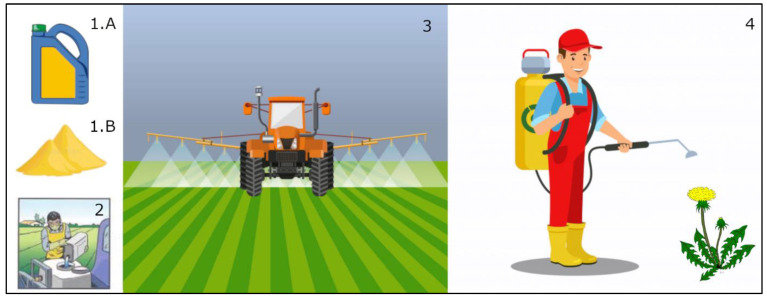
Illustration of risk for human health related to the use of EOs as bioherbicides. (**1.A**) Liquid product, (**1.B**) solid product, (**2**) filling, (**3**) application by tractor/cabin, (**4**) application by backpack sprayer with personal protective equipment.

First of all, the risk will depend on the physical state of the product used. EO-based bioherbicides can be liquid (Figure 1(1.A), solution or liquid encapsulation), but also solid (Figure 1(1.B), encapsulation in particles, adsorption on clay) [1].

For the user of a liquid EO-based bioherbicide, the major risk occurs, firstly, during the preparation and filling of the tank (Figure 1(2)) and, secondly, during the application of the product (Figure 1(3),(4)).

There is a risk of potential contact of the user’s hands with the product during the filling as well as in their respiratory system through inhalation of the volatile part during the whole use (preparation, filling and application). Whereas in the first case, it is considered as an acute herbicide poisoning risk, the second case is a chronical herbicide poisoning risk [57]. For both, the exposure risk to the users depends on the mode of application, e.g., tractor cabin (Figure 1(3), 85% hands) vs. backpack sprayer (Figure 1(4), head, arms, legs and feet exposed). The proper use of personal protective equipment will help to considerably decrease the dose the user would receive [1]. In addition, climate conditions (wind, temperature) during the use of the products will also influence this received dose. The combination of these exposures to the hazards presented in Section 2.1 allows us to conclude that depending on the major compounds of the EO used and the herbicide concentration, there is a risk of developing allergic contact dermatitis if the user does not protect their skin.

In many cases, this type of herbicide will induce the use of additives, wetting agents or encapsulating molecules, of which also their toxicities must be studied. For solid encapsulation of EOs, considerably lower exposure risks are found by preventing direct contact. In addition, as the release is slower, it will be less volatile.

Another risk is the development of mutation and/or cancer over the long term. However, this will depend on the quantity absorbed by inhalation of the user. This quantity is most likely too low to cause such a consequence since these harmful effects were only shown in in vitro experiments (higher concentration and direct exposure of cells to the product).

Take the example of an international patent for a bioherbicide based on essential oils (WO 2019/238948 A1) by conducting a critical analysis of their risks. This one is composed of 0.75% of *Cinnamomum cassia* EO (pre-emergence application) to 3.4% of *Cinnamomum cassia* or *Cinnamomum zeylanicum* EO (post-emergence application). In addition, these components promote the addition of an oily substance to the herbicidal composition, in order to decrease the volatility and improve the herbicidal effect. The study of the volatility showed that 200 mg of the herbicidal formulation (containing 3% of *Cinnamomum cassia* EO) is vaporized in 140 min at 20 ± 2 °C. It was specified that the cinnamon essential oil was composed of cinnamaldehyde, cinnamyl acetate, benzaldehyde, eugenol, eugenyl acetate, caryophyllene, linalool and phellandrene. Even if the relative concentration of each component can vary a lot, the first two components are the main components and represent, respectively, 33.9 to 76.4% and 0.09 to 49.63% [58].

In Table 1, we can see that cinnamaldehyde has mutagenicity and allergenic effects, with an LD50 of 2220 mg/kg for rodents, while cinnamyl acetate is not reported as toxic. The concentration of the herbicide formulation is highly under the value that induces an acute poisoning (indeed, it will need the user to be in contact with 100 L of the formulation to have dermal toxicity, and the quantity found by pulverization is too low (6 mg of EOs is vaporized in 2 h)). The risk for chronical exposure is still quite difficult to evaluate because this herbicidal formulation had not been experimented in the field, but once again the concentration absorbed will really be under the chronical poisoning concentration since spraying will occasionally be a pre-emergence application [59].

### 2.3. Risks for Human Health Related to the Drift in the Air

As EOs are a mixture of volatile compounds, a significant amount of the product could be drifted and, hence, transported through the air. However, the proportion of the herbicide lost by drift will depend on the formulation and the degree of encapsulation. As the use of EO-based bioherbicides is not yet common, there is no study of their impact on the air quality, carbone monoxide (CO) or ozone. However, the effects on evaporating EOs on indoor air quality have been widely studied. It has been proven that EOs reduce airborne microbial levels but increase CO, carbone dioxide (CO_2_) and volatile organic compound (VOC) levels, which could be problematic for human health [60]. Another point to be considered is the production of secondary pollutants such as formaldehyde following a reaction between oxidants and terpenes, and secondary organic aerosols in ozone [61].

### 2.4. Risks for Human Health Related to Residues in Feed

Nowadays, a major concern is the presence of herbicide residues in food, leading to the risk of chronic herbicide poisoning. Indeed, herbicides are used to protect crops before and after harvest from infestation by pests and plant diseases, but a negative consequence could be the presence of herbicide residues in feed. Although there have been multiple sources of contamination identified, direct herbicide deposition is the most important one.

However, also herbicide residues present in the soil and water are considered as critical elements of the herbicide cycle. The persistence of these residues depends on a wide range of factors, such as decomposition, volatilization, wind drift, runoff, chemical degradation, root uptake, leaching and microbial degradation [62,63].

In order to control the risk of chronical pesticide poisoning, the maximum residue level (MRL) for all crops and all pesticides has been integrated into the regulation for Good Agricultural Practice of the European Union [64]. Most countries in the world use MRLs to regulate herbicides, with an acute reference dose and the acceptable daily intake. They are different between countries and generally lower in Europe than in the US [31].

Here, the main advantage of using essential oils as bioherbicides is as follows: thanks to their volatility, the residues of EO components in food and soil are almost zero [38]. Conversely, this volatility induces the need to use a large amount of EOs which can have a negative impact on the environment and biota of the soil. Encapsulation is a good solution to find a balance between efficiency as a bioherbicide and not damaging the soil [8].

## 3. Conclusions

As it was stated at the beginning of this article, the natural origin of a molecule does not mean the absence of any hazard. Indeed, terpenes, terpenoids and phenolic compounds of an essential oil can be toxic, mutagenic and allergenic. Hence, this information must be considered when making use of them, depending on the dose. Indeed, it was shown in the risk analysis that the concentration of EOs used as bioherbicides is always much lower than the LD50 (at least 100 times less). As a result, the risk for acute poisoning is very weak. On the contrary, it is quite difficult to conclude the same for chronical poisoning because of the lack of information about the quantity and frequency of application in the field. Research must go further in the field of large-scale use of EO-based bioherbicides, and it is obvious that this will include the continuation of the above-mentioned patent [59].

The major benefit of the usage of EOs as bioherbicides instead of conventional herbicides is that the drastic reduction in the risk of air and soil pollution will be considerably lower, which may reduce the long-term negative impacts on human health.

This review focuses on human health risks of EO-based bioherbicides. The same approach can be easily applied to other related contexts where biopesticides are sprayed on crops as insecticides, bactericides or fungicides. However, it is important to consider the dose employed as well as the detailed composition of each EO used, which is, of course, different from that presented here in the context of herbicidal properties.

## Figures and Tables

**Table 1:** Major hazards for human health of compounds and EOs. Molecules with herbicidal activities are underlined.

Hazard	Compounds (Oral LD50 for Rodents, mg/kg)	EOs	References
**Cytotoxicity**	Eugenol (2680), isoeugenol, safrole methyleugenol (1179), estragole, 1,8-cineole (3849)	*Laurus nobilis* *Melaleuca leucadendron* *Sassafras albidum* *Ocotea pretiosa* *Ocimum basilicum* *Artemisia dracunculus* *Fusanus spicatus*	[35,38]
**Mutagenicity**	menthone, anethol (2090), asarone, trans-anethole oxide, trans-asarone oxide, terpineol (4300), cinnamaldehyde (2220), carvacrol, thymol (1800) and carvone (1640)	*Mentha Arvensis* *Pelargonium* *Pimpinella anisul* *Acorus* *Cinnamomum* *Thymbra capitata* *Thymus Vulgaris* *Carum carvi*	[42]
**Carcinogenicity**	Pulegone, safrole, methyleugenol, limonene (4600) and estragole	*Salvia sclarea* *Melaleuca quinquenervia*	[35,43]
**Allergenic effect**	Limonene (4600), linalool (>1000), estragole, phenyl acetaldehyde, methyl octinoate, citronellol, geraniol, benzyl alcohol, neral, geranial, α-isomethyl ionone, methyl eugenol (1179), hydroxy citronellal, α-ionone, eugenol (2680), cinnamaldehyde (2220), vanillin, coumarin, benzyl benzoate, benzyl salicylate, benzyl cinnamate	*Malaleuca alternifolia* *Cananga odorata* *Lavandula* *Mentha x piperita* *Pelargonium* *Rosa damascena* *Pistacia terebinthus* *Santalum album*	[44,45]
**Phototoxicity**		*Citrus aurantium dulcis* *Cymbopogon citratus*	[46]
**Reproductive toxicity**	Anethole (2090), apiole, citral (4960), camphor, thymoquinone, trans-sabinyl acetate, methyl salicylate, thujone, pulegone, β-elemene, β-eudesmol and costus lactone	*Pelargonium* *Petroselinum* *Anethum graveolens* *Cymbopogon* *Cinnamomum camphora* *Nigella sativa*	[47]
